# Selectivity of afferent microstimulation at the DRG using epineural and penetrating electrode arrays

**DOI:** 10.1088/1741-2552/ab4a24

**Published:** 2019-12-13

**Authors:** Ameya C Nanivadekar, Christopher A Ayers, Robert A Gaunt, Douglas J Weber, Lee E Fisher

**Affiliations:** 1 Department of Bioengineering, University of Pittsburgh, Pittsburgh, PA 15213, United States of America; 2 Rehabilitation Neural Engineering Laboratories, 3520 Fifth Avenue, Suite 300, Pittsburgh, PA 15213, United States of America; 3 Center for Neural Basis of Cognition, Pittsburgh, PA 15213, United States of America; 4 Department of Physical Medicine and Rehabilitation, University of Pittsburgh, Pittsburgh, PA 15213, United States of America

**Keywords:** neuroprosthetics, dorsal root ganglia, selectivity, somatosensory

## Abstract

**Objective.:**

We have shown previously that microstimulation of the lumbar dorsal root ganglia (L5-L7 DRG) using penetrating microelectrodes, selectively recruits distal branches of the sciatic and femoral nerves in an acute preparation. However, a variety of challenges limit the clinical translatability of DRG microstimulation via penetrating electrodes. For clinical translation of a DRG somatosensory neural interface, electrodes placed on the epineural surface of the DRG may be a viable path forward. The goal of this study was to evaluate the recruitment properties of epineural electrodes and compare their performance with that of penetrating electrodes. Here, we compare the number of selectively recruited distal nerve branches and the threshold stimulus intensities between penetrating and epineural electrode arrays.

**Approach.:**

Antidromically propagating action potentials were recorded from multiple distal branches of the femoral and sciatic nerves in response to epineural stimulation on 11 ganglia in four cats to quantify the selectivity of DRG stimulation. Compound action potentials (CAPs) were recorded using nerve cuff electrodes implanted around up to nine distal branches of the femoral and sciatic nerve trunks. We also tested stimulation selectivity with penetrating microelectrode arrays implanted into ten ganglia in four cats. A binary search was carried out to identify the minimum stimulus intensity that evoked a response at any of the distal cuffs, as well as whether the threshold response selectively occurred in only a single distal nerve branch.

**Main results.:**

Stimulation evoked activity in just a single peripheral nerve through 67% of epineural electrodes (35/52) and through 79% of the penetrating microelectrodes (240/308). The recruitment threshold (median = 9.67 nC/phase) and dynamic range of epineural stimulation (median = 1.01 nC/phase) were significantly higher than penetrating stimulation (0.90 nC/phase and 0.36 nC/phase, respectively). However, the pattern of peripheral nerves recruited for each DRG were similar for stimulation through epineural and penetrating electrodes.

**Significance.:**

Despite higher recruitment thresholds, epineural stimulation provides comparable selectivity and superior dynamic range to penetrating electrodes. These results suggest that it may be possible to achieve a highly selective neural interface with the DRG without penetrating the epineurium.

## Introduction

By 2020, over 2.2 million people in the United States will be living with limb loss [1]. Approximately 65% of amputations affect the lower limbs [1]. Lower-limb amputations are commonly associated with mobility issues, decreased balance confidence, and falling [2]. In stark contrast to these statistics, the acceptance rate of prevailing lower-limb prostheses is below 50% [3]. While there have been important advances in the design and actuation of prosthetic limbs, these devices lack a means for providing direct sensory feedback and force the user to rely on visual feedback or infer information about limb state from pressure exerted on the residual limb by the prosthetic socket. This results in longer rehabilitation, diminished control of prostheses, and reduced adoption and use of these technologies [4].

Multiple recent studies have demonstrated that stimulation of peripheral nerves in the residual limbs of amputees can evoke naturalistic sensory percepts, referred to the amputated limb, even decades after amputation [5–9]. Recent studies using epineural nerve-cuff electrodes, which wrap around peripheral nerves, have demonstrated that it is possible to achieve a long-term stable interface with distal peripheral nerves in people with arm [6, 10] and leg [11] amputations. While the sensations evoked by stimulation through these electrodes were highly stable over multiple years, the selectivity of stimulation (i.e. the ability to evoke sensations in focal areas of the distal limb) was somewhat limited. These limitations become especially obvious when comparing the focality of sensations generated by epineural stimulation to what can be achieved by stimulating peripheral nerves with penetrating microelectrode arrays, which are inserted into the nerve. For example, microelectrode arrays implanted in the median and ulnar nerves of amputees can generate highly localized sensation in the fingertips and palm [12] of the phantom hand, whereas nerve-cuff electrodes evoke sensations across multiple phalanges and larger areas of the palm and dorsum of the phantom hand [13]. Longitudinal intrafascicular electrodes implanted through peripheral nerves in individuals with upper limb amputation have also demonstrated the ability to evoke both focal proprioceptive and tactile sensations [9].

Unfortunately, there are a host of safety issues associated with implanting penetrating microelectrode arrays into neural tissue. Electrode insertion results in mechanical damage to the tissue followed by glial scarring [14] which isolates the electrode from the neurons [15], and in the periphery, shifts the fiber composition towards smaller fibers [16]. Additionally, peripheral nerves are highly stretchable structures that undergo large changes in length as the limbs move through their range of motion [17, 18], causing movement of the electrodes relative to their neural targets. All of these effects cause changes in the response to stimulation over time [19].

The dorsal root ganglia (DRG) and dorsal rootlets (DR) are attractive targets for delivering sensory feedback via electrical stimulation. The DRG contains a heterogenous population of cutaneous, muscle and nociceptive afferents all of which can be further divided into receptor classes that convey specific information about the state of the limb [20]. Three to four ganglia account for the innervation of an entire limb [21] while DRG at a single spinal level may provide access to the entire sensory representation of the foot [22]. Importantly, the separation of the sensory and motor pathway at the spinal roots allows for stimulation of afferents in the DRG without concomitant stimulation of motor efferents which could directly activate muscles and contaminate a myoelectric control interface. Additionally, the spine provides mechanical stability, limiting the movement of the DRG and spinal cord, which may improve the stability of stimulation. DRG and DR stimulation can also be used with high-level amputations (above-knee, above-elbow) where access to peripheral nerves is limited. In fact, ongoing work in our lab has demonstrated that electrical stimulation of the DR and spinal cord can evoke somatosensory percepts in the missing limbs of upper-limb amputees [23]. In that study, the stimulation electrodes were relatively large (3 × 1 mm), and stimulation evoked sensations that covered entire digits or regions of the palm. Stimulation through smaller electrodes might evoke substantially more focal sensations. Prior work in our lab has demonstrated that microstimulation via penetrating microelectrodes in the DRG can achieve a highly selective neural interface, recruiting many distinct distal branches of the sciatic and femoral nerves [24]. This would likely translate into focal percepts in the foot and leg in humans. However, in chronic experiments, we found a substantial degree of instability in the response to DRG microstimulation over time [25]. Additionally, these electrode insertion techniques require extensive exposure of the DRG (e.g. via foraminotomy), which may pose significant challenges for clinical translation.

One method of overcoming these disadvantages is to use non-penetrating epineural electrodes. Existing minimally invasive surgical techniques for implanting electrodes on or near the spinal cord and DRG to manage pain [26–28] can potentially be adapted for implanting epineural arrays on the DRG for sensory feedback. Clinically approved epineural stimulation leads [29–31] may be amenable to use for sensory feedback easing the clinical translation process and may provide a significant advantage over penetrating technologies. Additionally, our lab has recently demonstrated that it is possible to achieve single-unit recording of DRG neurons with electrodes placed on the epineural surface [32] which could be extended to study the mechanism of DRG stimulation or to develop a closed-loop neuroprosthesis that uses evoked responses to adjust stimulation parameters, similar to other currently available spinal cord stimulation systems [33].

Because it is challenging or impossible to have animals report on the characteristics of evoked sensations, nerve cuff recordings from multiple nerve branches have been used by our lab and others to measure the selectivity of peripheral nerve [34] and DRG stimulation [24]. In this study, we compare the recruitment properties of epineural and penetrating electrodes when stimulating afferents in the L5, L6 and L7 DRG. Electroneurographic recordings of evoked responses in many distal branches of the femoral (saphenous, vastus medialis, vastus lateralis, sartorius) and sciatic (tibial, distal tibial, medial and lateral gastrocnemius, common peroneal, distal common peroneal, sural, cutaneous) nerve were used to assess the selectivity of DRG stimulation. Compound action potentials (CAPs) recorded from each instrumented nerve were used to determine the threshold and dynamic range for selective recruitment, the distribution of projected fields per DRG, and the conduction velocity of the recruited afferents.

## Methods

All experiments were performed under the approval of the University of Pittsburgh Institutional Animal Care and Use Committee (IACUC) and the US Army Animal Care and Use Review Office. Acute experiments were performed in six anesthetized male cats.

## Methods: instrumentation

**Figure 1.**
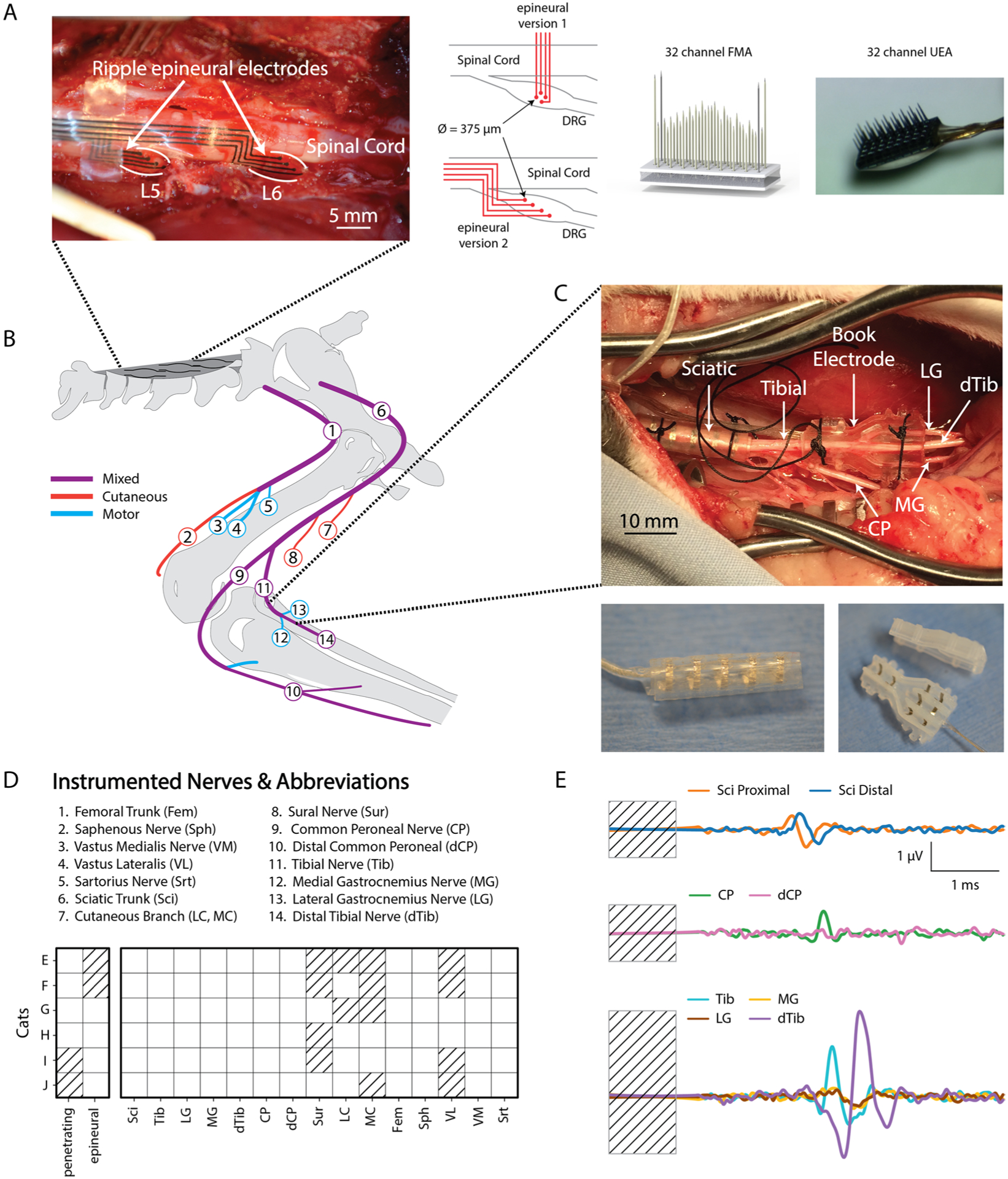
(A) Placement of Ripple stimulation electrodes on the epineurium of the L5 and L6 DRG (left) along with a representation of the 2 epineural electrode designs and images of 32-channel UEA and FMAs (right). (B) Schematic of nerve cuff location in the left hindlimb. (C) Nerve cuffs and book electrodes implanted on the sciatic trunk and distal branches. (D) Summary of nerves instrumented and DRG stimulation electrodes used across 6 acute experiments. Hatching represents instances where a nerve was not instrumented or a DRG stimulation electrode was not used. (E) Example stimulation triggered average ENG recorded from the sciatic nerve and its common peroneal (middle) and tibial (bottom) branches.

Anesthesia was induced with a ketamine/acepromezine cocktail and maintained via inhaled isoflurane (1%–2%) throughout the experiment. Vital signs (i.e. heart rate, core temperature, SpO_2_, and ETCo_2_) were monitored continuously. Distal branches of the femoral and sciatic nerves (figure 1(A)) were instrumented with two-contact nerve cuffs, which were either custom made or purchased (Microprobes, Gaithersburg, MD). Both types of electrodes were made from split silicone tubing with circumferential fine-wire stainless steel electrodes with an interelectrode spacing of 3 or 4 mm. The nerve cuff inner diameters ranged from 1 mm to 3 mm depending on the size of the targeted nerve. The sciatic and femoral nerves were instrumented with five-contact nerve cuffs (Ardiem Medical, Indiana, PA), which had an interelectrode spacing of 4 mm. Proximal, center, and distal contacts were shorted together and were used as a reference in a virtual tripole configuration when recording from the second and fourth contacts within the cuff [35].

Custom book electrodes were designed to match the characteristic branching pattern at the tibial nerve where it branches into the distal tibial, medial gastrocnemius and lateral gastrocnemius nerves (figure 1(B)). A 3D printed negative mold of this branching geometry was used to fabricate the spine and cover of the book electrode. Each channel in the book electrode contained two stainless steel electrodes to mimic a two-contact nerve cuff implanted on each branch.

Where possible, nerves projecting to members of each major muscle group innervated by the sciatic and femoral trunks were instrumented. The sciatic branches innervating the hamstrings were often very proximal, complicating surgical access, although a cuff was implanted around the nerve innervating biceps femoris in cat G. It was not possible to instrument the branch of the common peroneal nerve innervating ankle dorsiflexors without reflecting the biceps femoris tendon; however, the common peroneal nerve was always instrumented proximal and distal to this important branch point. Nerve identities were determined using known anatomical landmarks and verified by stimulation using a voltage-controlled stimulator (Grass, Warwick, RI) and finding coarse motor thresholds (supplementary table 1 (stacks.iop.org/JNE/17/016011/mmedia)). Sensory nerves, such as the sural and the sciatic cutaneous branch, were tested to the maximum stimulation intensity (20 V, 200 *μ*s pulse width) to verify that there were no evoked movements. Across the six cats, instrumented nerves included the Sciatic (Sci) and Femoral (Fem) trunks, lateral gastrocnemius (LG), medial gastrocnemius (MG), distal tibial (dTib), common peroneal (CP), distal common peroneal (dCP), sural (Sur), cutaneous branches of the sciatic nerve (Cut), saphenous (Sph), vastus lateralis (VL), vastus medialis (VM) and Sartorius (Srt) nerves (figure 1(D)).

After nerve cuff implantation, the left L5, L6 and L7 DRG were exposed via laminectomy. Epineural electrodes (4-channel Ripple LLC, Salt Lake City, UT) were placed on the epineurium of the L5, L6 and L7 DRG of cats G, H, I and J (figure 1(C)). For cats G and H, there was no fixation of electrodes to the epineural surface and a threshold search was repeated for multiple placements of a single array on multiple ganglia. For cats I and J the epineural electrodes were fabricated with tabs that were used for fixation to the spinal cord dura. Following epineural testing, penetrating arrays were implanted in the L5-L7 DRG and threshold search was repeated. Testing epineural arrays first limited the effects of surgical manipulation and the tissue damage that might occur during high-speed insertion of the penetrating arrays.

Penetrating floating microelectrode arrays (32-channel FMA; Microprobes, Gaithersburg, MD) were inserted in the L6 and L7 DRG of cats E and F. The platinum-iridium electrodes of each FMA (figure 1) had a variety of lengths (0.7–2.1 mm) designed to span the depth of the DRG with a pitch of 400 *μ*m and exposed tip sizes of 50 or 150 *μ*m. Utah electrode arrays (32-channel UEA; Blackrock Microsystems, Salt Lake City, UT) were inserted in the L5, L6 and L7 DRG of cats G and H. Each UEA contained 32 electrodes in a 4×8 grid and electrodes were 1 mm long with a pitch of 400 *μ*m. During implantation, a custom vacuum holder attached to a micromanipulator was used to position the array over the DRG. The array was positioned so that its long axis was aligned with the proximal/distal axis of the spinal root. A pneumatic inserter with 1.5 mm of travel (Blackrock Microsystems, Salt Lake City, UT) was used to rapidly insert the array through the epineurium in the DRG. For all electrodes, a stainless-steel screw in the iliac crest was used as the return for stimulation and all stimulation was applied in a monopolar configuration. The cat was placed in a spinal frame for the duration of the experiment. Motor thresholds were measured again after transfer to the frame to verify that the cuffs still made adequate contact and that the instrumented nerves were still intact.

## Methods: epineural electrode design

The epineural electrode array fabrication process was based on patterned robotic deposition of alternating layers of insulating medical-grade silicone/polyurethane co-polymer and a conductive polymer. The conductive polymer traces and electrode sites were formed by mixing platinum microparticles with the silicone/polyurethane substrate material. This provided mechanical matching of all the materials throughout the device for high flexibility and flexural durability. The flexibility of these electrodes allowed for conformation to the surface of the DRG. The initial design of these arrays, used in cats G and H, comprised of traces along the length of the array that terminated in four contacts arranged in a square layout (figure 1(A)). This electrode design was highly susceptible to mechanical perturbations when placed on the epineural surface. For cats I and J the array design was modified such that the leads from the array ran parallel to the spinal cord, providing additional friction with the epidural surface of the spinal cord to improve mechanical robustness. For these arrays, the traces terminated in four contacts arranged along the length of the DRG. Additionally, tabs were added to the array substrate to allow fixation to the spinal cord dura. The diameter of the exposed electrode contacts for both electrode arrays was 375 *μ*m and spacing between the centers of neighboring contacts was 750 *μ*m (mean ± std post-implant impedance was 16.03 ± 5.29 kΩ).

## Methods: experiment design

The objective of this study was to evaluate the recruitment properties of epineural electrodes in terms of threshold, selectivity, dynamic range, and distribution of recruited nerves. Threshold charge was defined as the minimum charge injection at the DRG required to elicit activity in any instrumented nerve. If a single distal nerve branch was activated at threshold, stimulation was deemed to be selective for that nerve. For each instance of selective recruitment, the dynamic range was determined as the range of stimulation charge over which selectivity could be maintained before a second nerve was recruited. In the event of non-selective recruitment, functionally synergistic innervation pathways (e.g. nerves innervating multiple heads of the gastrocnemius) were identified.

Electroneurogram (ENG) signals were recorded from all nerve cuffs using a Grapevine Neural Interface Processor (Ripple, Salt Lake City, Utah), using a differential headstage (Surf-D) with an input range of 5 mV, resolution of 0.2 *μ*V, 0.3 Hz cutoff high-pass filter and 7.5 kHz cutoff low-pass filter. Signal digitization was performed directly on the headstage at 30 kHz. Stimulation was performed using two IZ2 16-channel stimulus isolators (TDT, Alachua, FL) and custom LabVIEW software in cats E, F, G and H or Nano 2+Stim headstages (Ripple, LLC) for cats I and J. ENG signals typically have a low signal-to-noise ratio. To reduce this noise and reveal the underlying compound action potential (CAP), high pass filtering and stimulus triggered averaging was performed for all ENG recordings. Stimulation artifacts were blanked in software using a 1 ms window, which was at least 0.5 ms longer than each stimulation pulse and did not exceed the minimum conduction latencies of the most proximal nerves. Following blanking, ENG data were high-pass filtered at 300 Hz. Custom software was written in C++ and MATLAB (Mathworks, Natick, MA) to capture and display stimulus triggered ENG recordings from all cuff electrodes, to detect responses, and to coordinate a binary search for threshold as a function of the injected charge.

The methodology for determining recruitment threshold online is detailed elsewhere [24]. Briefly, a high amplitude survey trial was conducted to identify electrodes that evoked CAPs in the sciatic and femoral nerve branches. During this survey, stimulation was delivered through each electrode at a rate of 55–58 pulses s^−1^ with either 82 or 205 *μ*s/phase cathodic-leading symmetric pulses. The maximum charge injection for epineural stimulation was varied between 15–60 nC/phase (table 1). For penetrating electrodes, the maximum charge injection was varied between 3–8 nC/phase (table 2). This maximum amplitude was chosen to avoid electrode degradation and to avoid activating spinal reflexes that would cause muscle contraction and movement artifact in the ENG signal. Stimulation electrodes that did not evoke a response in any nerve branch were excluded from the binary search. For all other electrodes, a binary search over stimulation charge was carried out to determine the recruitment threshold for each instrumented nerve. For epineural electrodes, the resolution for the binary search was varied between 0.06 and 0.95 nC/phase (table 1) and for penetrating electrodes the resolution was varied between 0.17 and 0.71 nC/phase (table 2). The binary search resolution used for epineural testing was lower relative to penetrating electrode testing (i.e. stimulation charge was sampled more coarsely) to compensate for the higher survey trial amplitude. For cat G, the binary search with penetrating electrodes at L5 DRG and epineural electrodes at L7 was conducted at two separate resolutions within the same experiment to reduce the time spent performing a binary search per electrode. A non-parametric Kruskal-Wallis test confirmed that resolution had no effect on the detected threshold (*p* > 0.01) for cat G. During the online threshold search, stimulation was repeated 400–600 times at each amplitude, ENG responses were detected by comparing the RMS of the stimulus triggered averaged ENG response between pre-stimulus baseline and stimulation epochs. The windowed RMS was calculated using a 250 *μ*s sliding window with 25 *μ*s overlap between consecutive windows. RMS values exceeding 0.5 *μ*V and one standard deviation of baseline RMS for four consecutive windows were annotated as stimulation evoked responses. These param eters were selected empirically to improve accuracy during online detection.

All recorded ENG signals were reanalyzed offline for rigorous statistical testing using a non-parametric subsampling approach described previously [24]. Briefly, a 99% confidence interval about the baseline mean was established and the detection threshold for post-stimulation ENG responses was set to one standard deviation above the upper bound of this interval. For each stimulation amplitude, a random subsample of 90% of the repetitions were selected 100 times to generate a distribution of ENG responses for each electrode. The windowed RMS was calculated for each of these subsampled responses using the same 250 *μ*s sliding window with 25 *μ*s overlap. For a time-window in the ENG response to be considered significant, 95% of the subsampled averages had to be supra-threshold during that time window (supplementary figure 1(A)). Additionally, all ENG responses from epineural stimulation were validated by a human expert. The sensitivity and accuracy of the automated detection algorithm was calculated using the manual annotations as ground truth. ENG responses per trial for both epineural and penetrating electrodes along with validated annotations can be downloaded from the Blackfynn Discover Repository at https://doi.org/10.26275/emdv-q3jm. Additionally, all ENG responses and selectivity results per trial can be viewed at https://lumbarselectivity.herokuapp.com.

## Methods: conduction velocity

For instances where a CAP was detected at the sciatic or femoral nerve trunks, the local cross correlation (LCC) was calculated between signals recorded from the second and fourth contacts of the 5-pole nerve-cuff to determine the conduction velocity of recruited afferents. The process for calculating LCC is described in detail elsewhere [35]. Briefly, the cross-correlation between the stimulus-triggered averaged signal recorded on the second contact and fourth contact was calculated (supplementary figure 1(B)). A 0.5 ms sliding window of the signal recorded from the fourth contact was moved through a 1–10 ms time window of the stim-triggered average ENG signal recorded on the second contact at 50 *μ*s steps. If the peak of the LCC exceeded one standard deviation above the cross-correlation of the noise for three consecutive windows, the trial was identified as containing a compound action potential. The distance between the second and fourth contact (8 mm) was divided by the cross-correlation lag for the window with the highest LCC to calculate the conduction velocity of the recruited afferent.

## Results

Across the four cats (G, H, I, J) where epineural stimulation was delivered, a total of 64 electrodes were tested in 11 ganglia. Fifty-two electrodes produced a response in at least one nerve at threshold and 67% of these electrodes were able to selectively recruit a single nerve at threshold. In contrast, for the four cats (E, F, G, H) where penetrating arrays were tested, a total of 672 electrodes were tested in 10 ganglia, of which 308 produced a response at maximum amplitude and 79% of these electrodes selectively recruited a single distal nerve branch at threshold. While the percentage of responsive electrodes evoking selective responses were higher for penetrating arrays, stimulation at maximum amplitude evoked responses in fewer penetrating electrodes (45%) compared to epineural electrodes (76%). The percentage of responsive electrodes varied across subjects for both electrode types. For penetrating electrodes, the yield of responsive electrodes at maximum amplitude for cats E, F, G, and H was 31.7%, 14.8%, 96.0%, and 89.6% respectively. For epineural electrodes the yield for cats G, H, I, and J was 50%, 95.8%, 91.7%, and 66.7%. The maximum amplitude delivered during epineural stimulation was higher than that for penetrating stimulation and may explain overall greater recruitment.

## Results: coactivation at threshold

**Figure 2.**
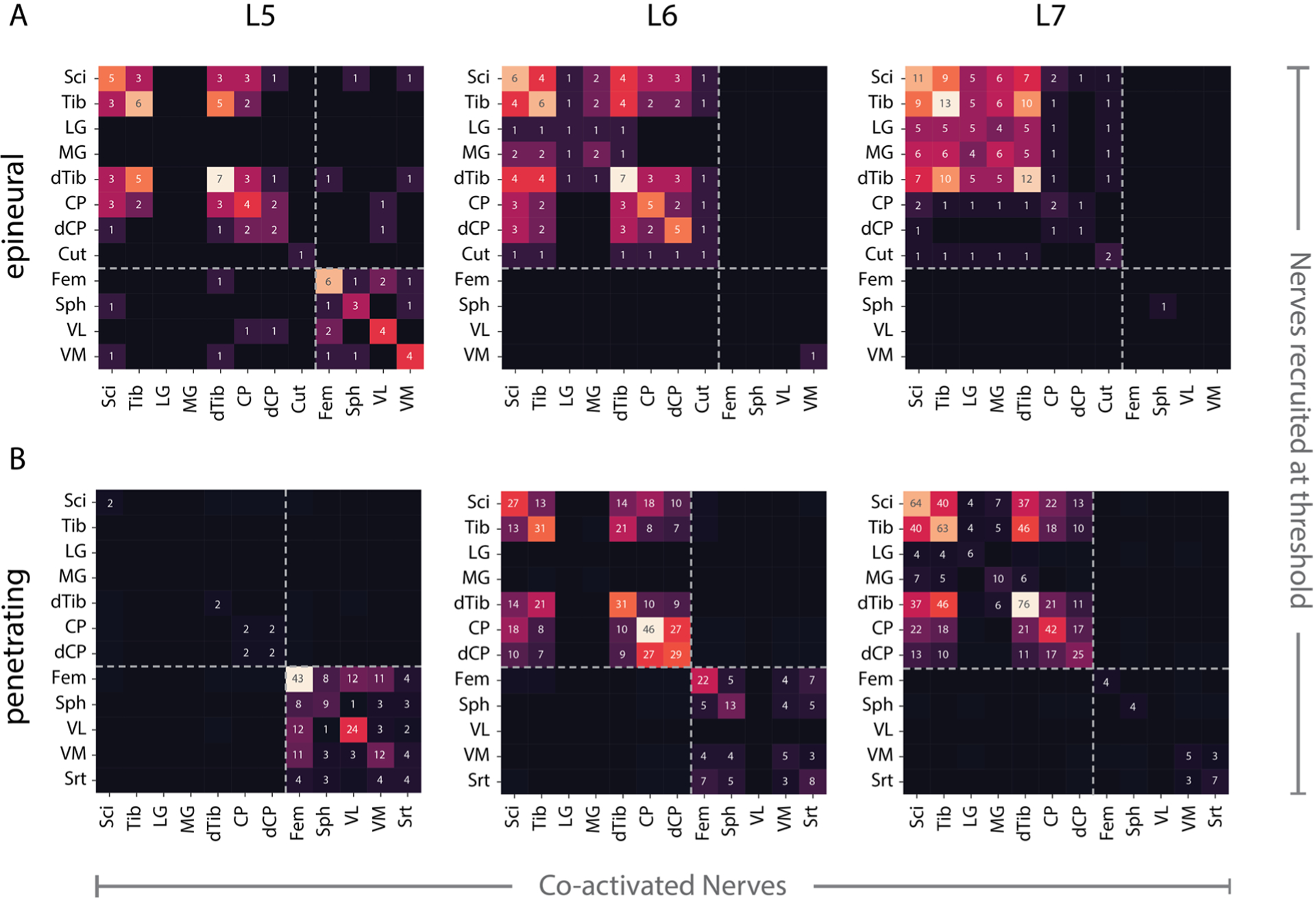
Count of nerves coactivated at threshold per DRG for stimulation via (A) epineural and (B) penetrating electrodes. Dashed line indicates division between femoral and sciatic nerve branches. Rows represent the nerves recruited and columns represent the nerves coactivated at threshold.

Threshold responses for stimulation at each DRG were used to generate coactivation matrices for each electrode type (figure 2). The rows in each coactivation matrix correspond to the nerve recruited at threshold and the columns represent the coactivated nerves. The non-normalized counts for recruitment and coactivation were calculated to highlight the differences in recruitment and coactivation per DRG. Additionally, non-normalized counts allow comparison across multiple nerves recruited at threshold whereas normalizing (supplementary figures 2(A) and (B)) allows a comparison of the instances of coactivation within a given nerve. With epineural electrodes, stimulation at the L5 DRG recruited femoral and sciatic nerve branches at threshold. Stimulation at the L6 and L7 DRG exclusively recruited the sciatic nerve and its branches at threshold except for one instance of vastus medialis recruitment at L6 and one instance of saphenous recruitment at L7. Epineural stimulation at L6 recruited the common peroneal and the tibial branch of the sciatic nerve, however LG and MG were rarely recruited. Whereas stimulation at L7 preferentially recruited the tibial branch of the sciatic nerve over the common peroneal branch, and had a higher recruitment rate for MG and LG. With penetrating electrodes, stimulation at L5 primarily recruited the femoral trunk and its branches (Sph, VL, VM and Srt) with minimal sciatic nerve recruitment. Penetrating electrode stimulation of the L6 DRG recruited sciatic and femoral branches approximately equally at threshold with no activation of the LG, MG and VL. While penetrating L7 stimulation predominantly recruited sciatic nerve branches with rare activation of LG and MG, unlike epineural stimulation at L7. Both electrode types demonstrated preferential activation of sciatic nerve branches for stimulation at L6 and L7 DRG. However, stimulation with penetrating electrodes at the L6 DRG showed more coactivation of sciatic and femoral branches than epineural stimulation. This trend was reversed for stimulation at the L5 DRG where epineural stimulation produced more coactivation of femoral and sciatic nerve branches at threshold than stimulation with penetrating electrodes. The combined coactivation matrices for each electrode type (supplementary figures 2(A) and (B)) were calculated by adding the coactivation matrix at each DRG and normalizing the counts in each row by dividing by the total number of times that a given nerve was recruited. This was used to determine the overall tendency of multiple nerves to be coactivated at threshold. The overall pattern of coactivation at threshold for penetrating and epineural electrodes showed a strong linear relationship (supplementary figure 2(C)) i.e. the likelihood of two nerves being coactivated at threshold was similar for both electrode types. The Pearson correlation coefficient between the coactivations for epineural and penetrating stimulation was 0.80 (*r*^2^ = 0.64). We expected that distal nerves would be coactivated with their proximal parents (e.g. tibial or common peroneal with sciatic nerve). However, this was not always true and is likely a result of the difficulty in detecting threshold-level ENG signals in large-diameter nerves, where the signal may be smaller if the electrode is further from the source [24]. Furthermore, the sciatic and femoral nerves were infrequently coactivated at threshold (<20% of the time).

## Results: selectivity and dynamic range

**Figure 3.**
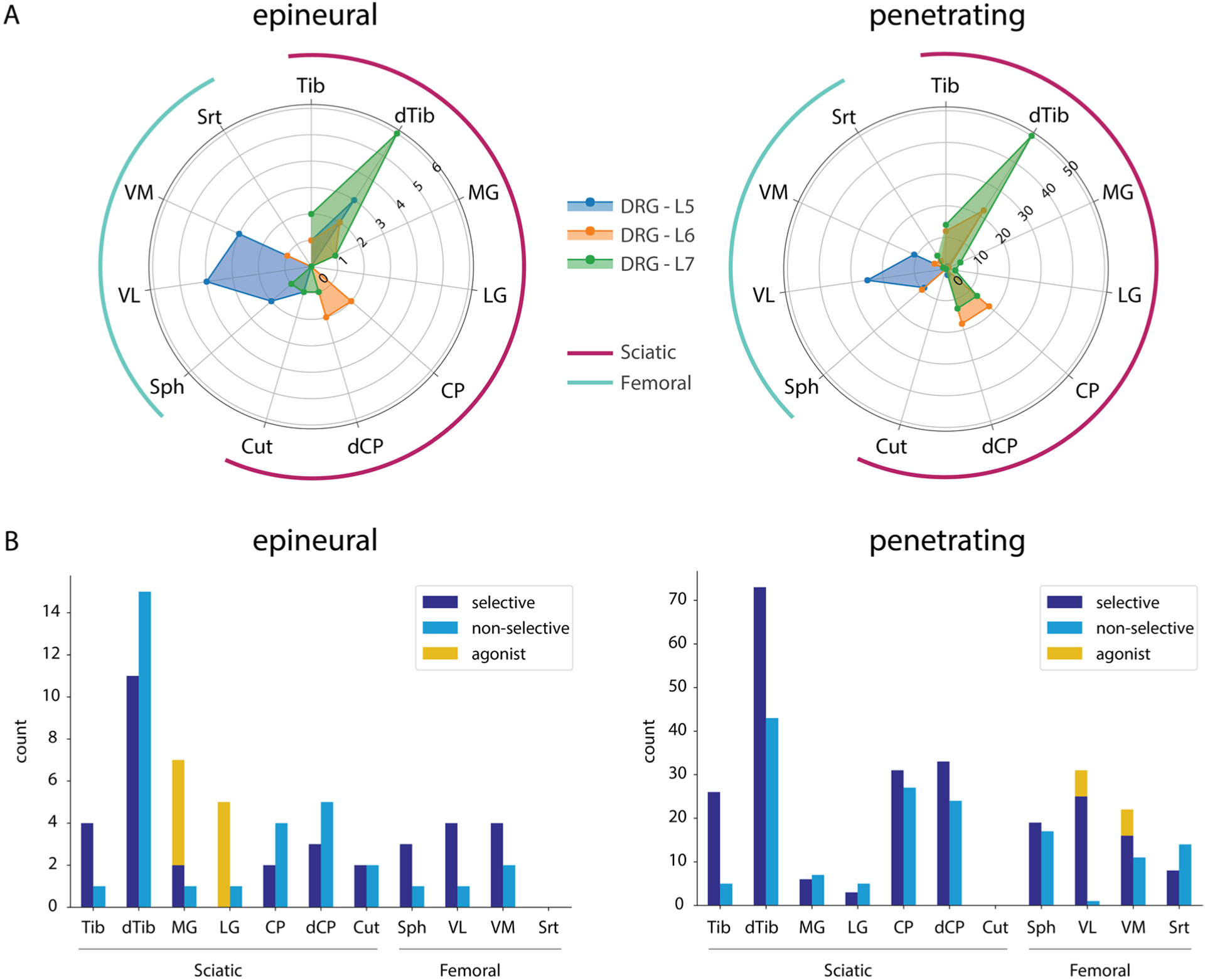
(A) Counts of selectively recruited nerves for stimulation via epineural and penetrating electrodes at each DRG. (B) Counts for selective recruitment, non-selective recruitment and coactivation of agonists at threshold. Counts for selective recruitment were obtained by adding the counts at each DRG for each nerve in (A). Nerves in the same innervation path (e.g. tibial and distal tibial) were allowed to be coactivated while still being considered selective; however, only activation of the distal most nerve was counted to highlight differential recruitment of proximal branches.

The instances of selective recruitment for each nerve were tallied for each DRG (figure 3(A)). Stimulation that recruited a single distal nerve at threshold was deemed selective. Nerves in the same innervation path (e.g. tibial and distal tibial) could be coactivated while still being considered selective. However, only activation of the most distal nerve was counted to highlight differential recruitment of proximal branches [24]. Epineural stimulation at the L5 DRG recruited more sciatic branches (28%) than penetrating stimulation (6%). For both electrode types, the distal tibial nerve was most often recruited selectively. This was followed by the tibial, vastus lateralis, and vastus medialis branches for epineural stimulation and distal common peroneal and common peroneal branches for penetrating stimulation. For both electrode types, stimulation at the L5 DRG produced selective responses in the femoral nerve and its branches. For the L6 and L7 DRG, the likelihood of recruiting the sciatic nerve or its branches was higher than for L5. Additionally, for functional groups of agonist muscles, (i.e. quadriceps: VL, VM and plantarflexors: LG, MG), selective recruitment of each nerve and each functional agonist group was counted separately (figure 3(B)). For epineural stimulation, the quadriceps were never coactivated at threshold while the plantarflexors were often (55%) coactivated at threshold. Overall, epineural stimulation yielded at least one instance of selective recruitment at threshold for all the instrumented nerves except LG. For penetrating electrodes, plantarflexors (MG and LG) were never coactivated at threshold while the quadriceps were coactivated infrequently (8.3%).

Using an expert observer as the gold standard, the overall sensitivity, specificity, and accuracy for ENG response detection was 96.4%, 80.9% and 85.7%, respectively (supplementary table 2). In several instances, the ENG response detection algorithm erroneously detected the stimulation artifact as an ENG response, which was greater in the femoral nerve trunk due to the short distance between the DRG and the femoral nerve cuff. This high false positive rate (Fem: 30%, Sci: 3%) contributed to the lower detection accuracy in the femoral nerve and its branches, particularly for cats G and H.

**Figure 4.**
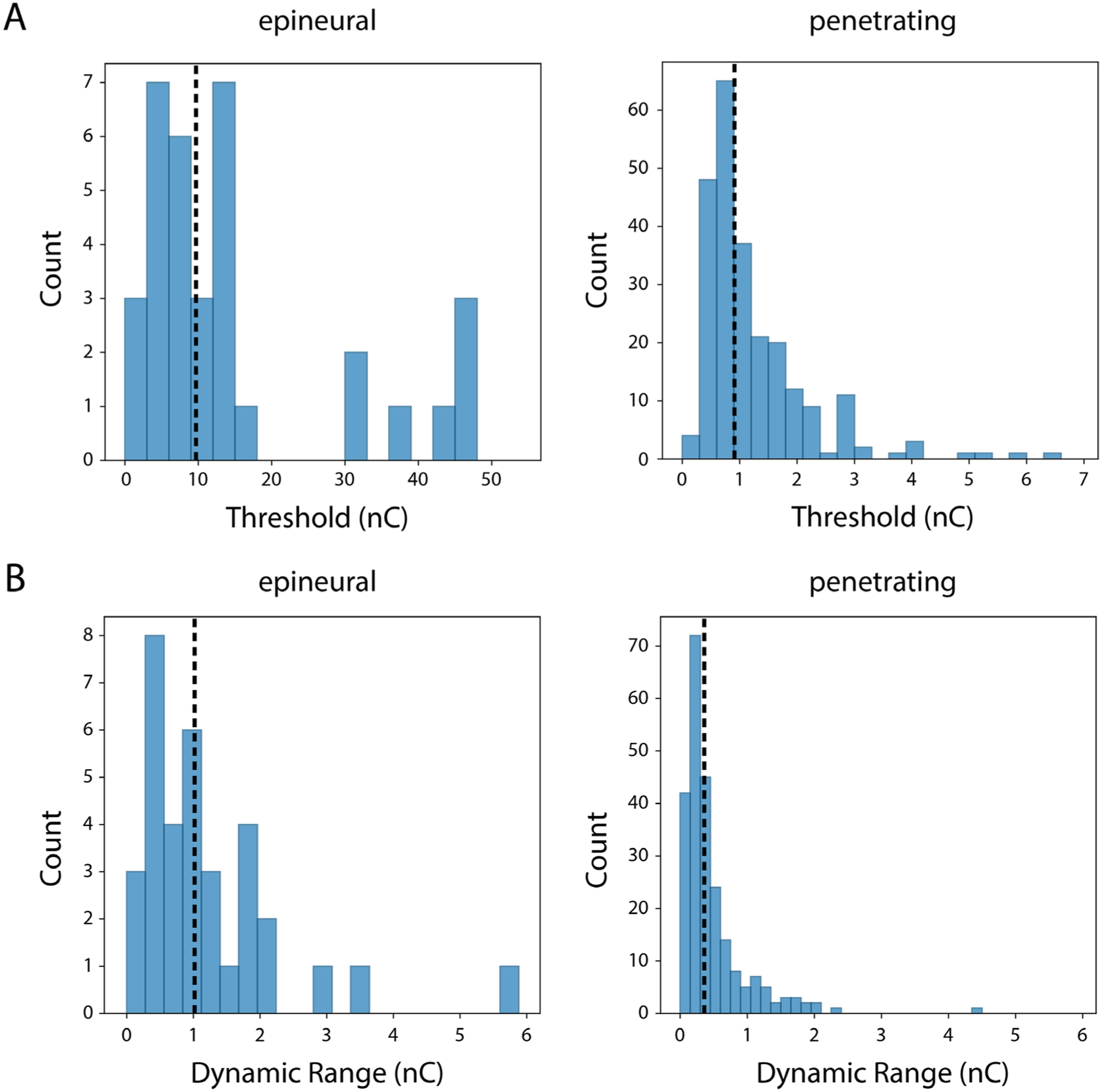
(A) Distribution of selective recruitment thresholds for stimulation via epineural (median = 9.67 nC/phase) and penetrating (median = 0.905 nC/phase) electrodes. B) Distribution of dynamic range for selective recruitment for epineural (median = 1.01 nC/phase) and penetrating (median = 0.36 nC/phase) stimulation. Dashed lines show median for each plot.

We also quantified the distribution of threshold charge and dynamic range of stimulation for each instance of selective activation. A D’Agostino-Pearson test was used to determine that the distributions for threshold and dynamic range for each electrode type (figure 4) were non-normal (*p* < 0.01), so a non-parametric Kruskal-Wallis test was used to test for differences in thresholds and dynamic range across epineural and penetrating stimulation. The median recruitment threshold and dynamic range for epineural electrodes were 9.67 nC and 1.01 nC, respectively. For penetrating electrodes, median recruitment threshold and dynamic range were 0.90 nC and 0.36 nC, respectively. Both threshold and dynamic range were significantly higher (*p* < 0.001 for both) for epineural than penetrating electrodes. The choice of resolution for a binary search could impact the dynamic range (i.e. a lower resolution could give the appearance of a higher dynamic range). Since the resolution was varied between epineural and penetrating electrode experiments, we used a non-parametric Kruskal-Wallis test to test for differences in dynamic range across binary search resolutions for each electrode type (supplementary figure 3). For the same binary search resolution (0.41), there were significant differences (*p* < 0.01) in the distribution of the dynamic range for penetrating and epineural stimulation. Additionally, the dynamic range for penetrating stimulation at a lower resolution of 0.082 nC was not significantly different from the dynamic range detected with a resolution of 0.41 and 1.024 nC with epineural electrodes. These results indicate that the observed differences in dynamic range were not due to the resolution of the binary search.

Additionally, we used a nonparametric Kruskal-Wallis test (as data were non-normal) to test for differences in dynamic range and threshold between FMA and UEA penetrating electrodes. There were significant differences in the thresholds (*p* < 0.01) between the two types of penetrating electrodes, however no difference was observed in the distribution of dynamic range. We also tested if there were differences in threshold and dynamic range across electrode length and exposed tip sizes for the FMAs and no significant differences were observed. We also tested for differences between the two designs of epineural electrodes used and found a significant difference in the thresholds (*p* < 0.01). However, there was no difference in the dynamic range between both designs. Because of variations in the experimental setup between cats G–H and I–J, there were multiple covarying factors that could have led to these differences such as the layout of contacts (linear versus square) and fixation to the spinal dura.

## Results: conduction velocity

**Figure 5.**
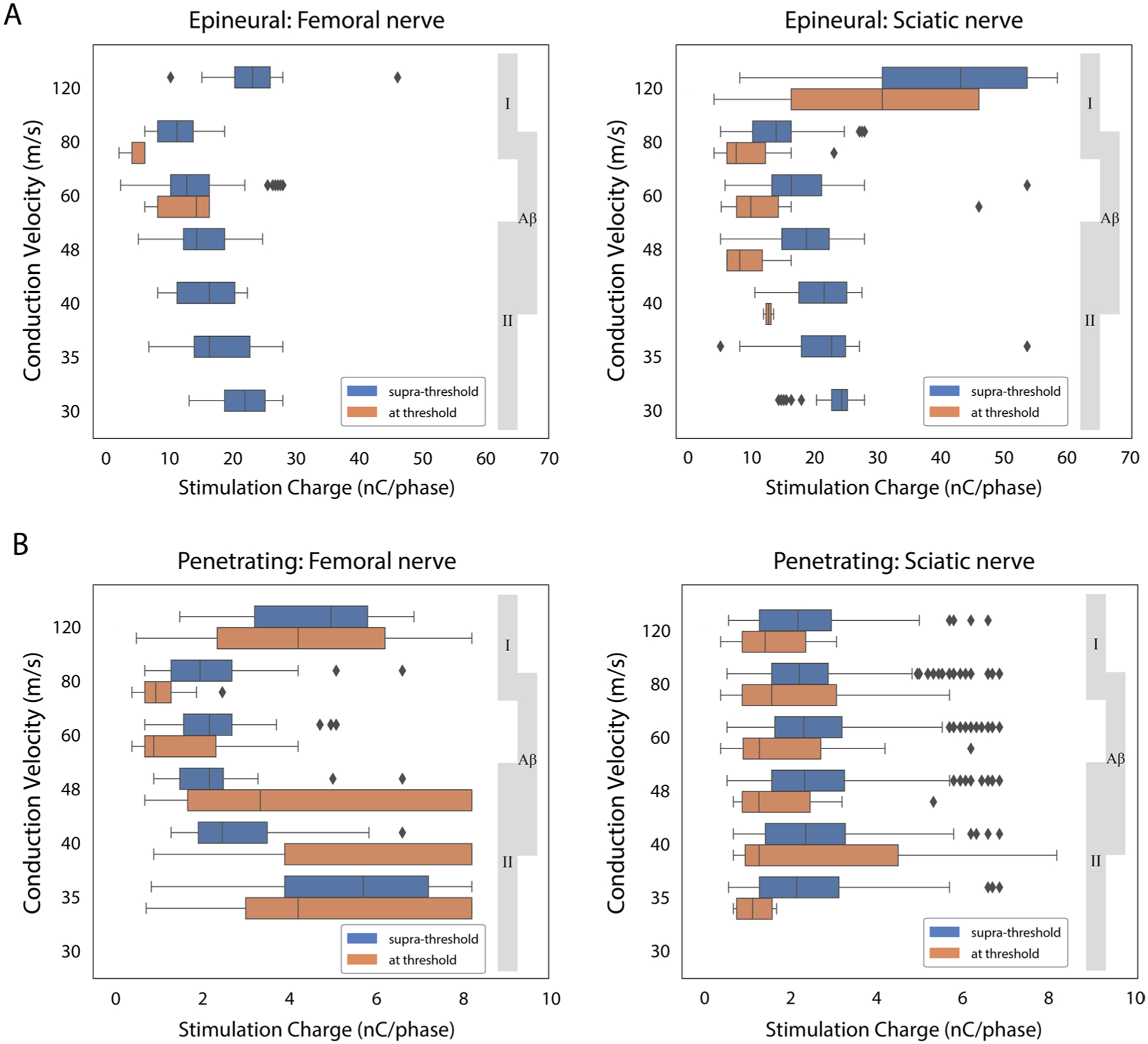
Comparison of stimulation charge injected and the CV of the nerve cuff response recorded across all stimulation amplitudes (blue) and at recruitment threshold (orange) for stimulation via (A) epineural and (B) penetrating electrodes at the lumbar DRG. CV values were discretized due to sampling frequency limitations of the recording setup. The vertical grey bars indicate the range of CVs corresponding to group I, A*β* and II afferents.

Finally, the conduction velocity of recruited afferents in the femoral and sciatic nerve were compared at threshold and supra-threshold stimulation amplitudes (figure 5). For epineural stimulation, at threshold only 60–80 m s^−1^ (group 1/A*β*) fibers were recruited in the femoral nerve while slowly conducting fibers (<50 m s^−1^, group 2) were recruited at higher amplitudes. For the sciatic nerve, epineural stimulation recruited 40–120 m s^−1^ fibers at threshold while slowly conducting fibers were never recruited at threshold. The relationship between stimulation amplitude and conduction velocity of recruited fibers displayed a weak negative correlation at threshold for the femoral nerve (*R*^2^ = 0.39, slope = −2.26 m s^−1^ nC^−1^, *p* < 0.01) and a weak positive correlation for the sciatic nerve (*R*^2^ = 0.35, slope = 1.19 ms^−1^ nC^−1^, *p* < 0.001). For penetrating stimulation, the range of fibers (35–120 m s^−1^) recruited at threshold and supra-threshold stimulation amplitudes was the same for sciatic and femoral nerves. Stimulation via penetrating electrodes did not recruit any afferents with a conduction velocity below 35 m s^−1^. For the femoral nerve, the conduction velocity of recruited afferents at threshold showed a weak negative correlation with the stimulation charge (*R*^2^ = 0.17, slope = −2.44 m s^−1^ nC^−1^, *p* < 0.001) whereas for afferents in the sciatic nerve, no linear correlation between stimulation amplitude and conduction velocity was observed.

## Discussion

The goal of this study was to determine whether epineural stimulation of the DRG selectively recruits distal branches of the sciatic and femoral nerves and to compare the distribution of recruited nerves and recruitment properties with stimulation via penetrating electrodes. While epineural electrodes provide a clearer path to clinical translation than penetrating devices, the epineurium is a resistive barrier [36] that increases the separation between electrode sites and target neural tissues. Additionally, the size of the active sites on the epineural electrodes used in these experiments meant that charge density delivered per pulse of stimulation was lower than with penetrating electrodes. Given the diffuse nature of epineural stimulation, we expected less selective recruitment and frequent coactivation of the sciatic and femoral branches. Surprisingly, a majority (67%) of epineural electrodes selectively recruited a single distal branch of the sciatic or femoral nerve at threshold.

The pattern of recruitment was consistent with known dermatome maps [21] and selectivity was consistent across both electrode types. Stimulation of the L5 DRG selectively recruited femoral nerve branches innervating the quadriceps while stimulation at the L6 and L7 DRG recruited sciatic nerve branches innervating ankle plantar flexors and distal branches of the common peroneal and tibial nerves, both of which innervate the skin on the foot. Several studies have demonstrated that sensory feedback from peripheral afferents is necessary to modulate locomotor muscle activity and the timing of phase transitions in the gait cycle [37–41]. Specifically, feedback from Golgi tendon organs and secondary spindle afferents provides approximately 1/3 of ankle plantar flexor muscle tone [42]. In the context of clinical translation, these results imply that delivering stimulation at the caudal lumbar DRG may be sufficient to evoke relevant percepts that are localized to the missing limb in a somatosensory neuroprosthesis for people with trans-tibial amputation.

Other than the lateral gastrocnemius nerve, epineural stimulation selectively recruited every instrumented nerve branch at least once. Interestingly, for both epineural and penetrating electrodes the distal tibial nerve was frequently recruited selectively. It is unlikely that distal tibial afferents have an intrinsically low activation threshold. However, it is possible that afferents innervating the distal tibial nerve represent a greater fraction of afferents than other nerves in the L6 and L7 DRG, increasing the likelihood of recruitment. While our primary method to determine selectivity was to identify nerves that were recruited to the exclusion of all others, another way to consider selectivity is at a functional level. For example, there were five instances in which the lateral and medial gastrocnemius nerves were coactivated at threshold. Although this coactivation does not represent selectivity in terms of a single nerve branch, both nerves innervate the agonist muscles that are responsible for ankle plantarflexion and knee flexion. In terms of delivering relevant sensory feedback, it may not be necessary to selectively recruit these functionally synergistic nerves. The same holds for the vastus lateralis and medialis nerves that innervate the quadriceps, although coactivation at threshold was not observed during epineural stimulation.

As anticipated, epineural stimulation had a higher threshold for selective recruitment when compared to penetrating electrodes. However, the dynamic range of selectivity was also significantly higher than that for penetrating electrodes. This means that selectivity for a given nerve could be maintained over a larger range of charge injection during stimulation. In the context of clinical translation, a higher dynamic range for selective recruitment may provide a somatosensory interface that is resilient to electrode encapsulation since increasing stimulation charge may still recruit the same population of afferents. A larger dynamic range also provides a larger parameter space within which stimulation can be varied in order to modulate the subjective quality of an evoked percept. The inherent assumption that epineural stimulation is diffuse may be true in the context of current spread through neural tissue. However, it is plausible that the mechanism of afferent recruitment itself is different between epineural and penetrating stimulation and serves to counter this diffusivity. Prior DRG modelling work has predicted the probability of recruiting a distribution of fibers as a function of stimulus intensity [43]. This model demonstrated that as stimulation amplitude increased the number of fibers recruited increased exponentially. Furthermore, it also predicted that medium-diameter fibers may be recruited with greater probability than large-diameter fibers. While these results formed the basis for our *in vivo* experimentation with penetrating stimulation electrodes, the model does not account for the presence of cell bodies, the pseudounipolar morphology of DRG afferents, the glomerular structure of the T-stem axon [44] and assumes that the site of activation always occurs at the nodes of Ranvier in axons.

Results from our epineural stimulation experiments may contradict this assumption about the site of activation within afferents in the DRG. When the DRG is viewed in cross-section, the cell bodies of afferents are clustered around the circumference, while axons are more densely located near the center of the structure [45]. This unique anatomical structure may explain the surprising selectivity results we observed. Epineural electrodes are much closer to cell bodies than to axons, and given that voltage drops exponentially with distance from an electrode and that epineural stimulation can achieve a high degree of selectivity, it is possible that the site of activation may not always be the axons.

Several studies modeling the behavior of neurons at the DRG have confirmed empirical observations regarding the excitability of the soma. The cell soma is adapted for spike initiation and the excitability of the soma is essential for spike invasion of the soma [46]. Our data presents the possibility that epineural stimulation at the DRG may activate other regions of neurons (e.g. axon hillock, stem), that are closer to the epineurium while penetrating electrodes directly recruit axons present in higher densities near the center of the DRG. Future work should explore these potential mechanisms and their implications for the design of electrodes at the DRG.

The subjective quality of evoked percepts may also be modulated by activating fibers of specific sensory modalities. Afferents can be loosely segregated into separate populations based on their axonal diameters and corresponding conduction velocities, though those populations have some overlap [47–49]. By measuring the conduction velocity of CAPs traveling through the nerve-cuff electrode, it is possible to infer the most likely sensory modalities of the activated neurons. Our prior work in acute and chronic preparations using penetrating electrodes has demonstrated the ability to recruit medium to large diameter fibers in the DRG [25, 35]. Additionally, modeling studies examining the recruitment of fibers via stimulation of the DRG have demonstrated that intraneural microstimulation may recruit medium diameter fibers with greater probability than large diameter fibers [43] while non-penetrating DRG stimulation drives activity of large myelinated A*β* fibers but does not directly activate small nonmyelinated C-fibers [50]. In the present study, recruitment of fibers in the L5, L6 and L7 DRG showed a similar trend. Across all stimulation amplitudes, epineural electrodes recruited fibers with conduction velocities between 30–120 m s^−1^. Stimulation with penetrating electrodes recruited a similar range of fibers (35–120 m s^−1^). These conduction velocities correspond to medium and large diameter fibers such as muscle spindle afferents, Golgi tendon organs and a variety of cutaneous sensory axons. At threshold, epineural stimulation recruited fibers in the femoral nerve with conduction velocities between 60–80 m s^−1^, roughly corresponding to medium diameter fibers. Interestingly, fast conducting (120 m s^−1^) fibers were not activated selectively at threshold. For the femoral nerve, stimulation via epineural and penetrating electrodes displayed a similar negative correlation between the conduction velocity of recruited afferents and the stimulation charge. For the sciatic nerve, epineural stimulation at threshold predominantly recruited fibers with conduction velocities between 40–80 m s^−1^ with some instances of 120 m s^−1^ fibers being recruited. As with the modeling study, this result may occur because of the higher percentage of cutaneous afferents than fast conducting proprioceptive afferents in the DRG [51]. In contrast, stimulation via penetrating electrodes recruited a diverse population of afferents projecting to the sciatic nerve at all stimulation amplitudes. Still, we have demonstrated that epineural electrodes can recruit a range of sensory afferents and future work may focus on selective recruitment and activation thresholds for each modality of sensory afferents with epineural DRG stimulation.

## Challenges and future work

In this study we have demonstrated that selective recruitment of distal nerve branches that innervate the hindlimb via epineural stimulation is comparable to the selectivity achieved via penetrating electrodes. Despite the diffuse nature of epineural stimulation, individual nerves and functionally synergistic innervation pathways were recruited at threshold and the dynamic range for selectivity was higher than anticipated. However, there are a few shortcomings that could be addressed in future work. A large inter-subject variability was observed in the percentage of epineural electrodes that produced a response at maximum charge injection. It is possible that this variability is an example of anatomical differences and inconsistent dermatomes between animals. However, successful array placement and fixation typically determined the efficacy of stimulation. There was no fixation of electrodes to the epineural surface for cats G and H while epineural electrodes used with cats I and J were fabricated with tabs on the arrays that were used for fixation to the spinal cord dura. For a potential somatosensory interface with the DRG, ensuring electrode positioning and contact with neural tissue throughout the lifetime of the device is critical. Additionally, in terms of measuring conduction velocity, we were limited by the sampling frequency of the surf-D headstage (i.e. 30 kHz). This meant that the resolution for measuring conduction velocity decreased for higher conduction velocities and we could only detect conduction velocities at 30, 34, 40, 48, 60, 80 and 120 m s^−1^. While no afferents were recruited below 40 m s^−1^ it is possible that the reported conduction velocities are only a close approximation (to the nearest sample) and selective recruitment by sensory modality may be possible.

The difficulty of selectively recruiting neurons varies throughout the nervous system based on underlying neural organization. Neural interfaces based in the primary somatosensory [52, 53] and the visual cortex [54, 55] rely upon the somatotopy and retinotopy of these regions, respectively. The cochlear implant relies on the well-defined tonotopic map of the cochlea [56] which facilitates recruitment of auditory fibers with similar frequency responses in spatially restricted locations. While there is dermatomal segregation of afferents from large regions of the limb across DRG, selective recruitment is important due to the lack of a consistent somatotopic organization of sensory fibers within each DRG [57, 58]. As with peripheral nerves, neighboring neurons may innervate different regions within a dermatome or may convey different modalities of sensory information. This means that particular afferent types and subregions of the limb cannot be targeted *a priori* with DRG stimulation, making broad and diverse electrode coverage especially important. In the context of developing a clinical somatosensory interface, further work is necessary to evaluate the effect of modulating stimulation parameters on recruitment properties. In this study all stimulation was applied in a monopolar configuration with respect to a distant ground. Maximizing the utility of epineural electrodes may ultimately require pursuing bipolar stimulation and more complex current steering techniques to achieve more focal recruitment. The epineural electrodes used in this study had only four contacts. We tried multiple placements of these electrodes to compensate for this limitation. Additionally, the large electrode contacts meant multiple electrodes may have recruited redundant populations of afferents. The size and spacing of electrodes used in this study was constrained by the fabrication process. Ripple is currently able to manufacture devices with features of 150 *μ*m that are comparable in size to the cell body of afferents found in the DRG (20–100 *μ*m) [59]. Ongoing work in our lab is focused on identifying the optimal electrode size, channel count, spacing, and fixation required for maximal coverage, selectivity and electrode independence. The primary limiting factors for achieving a high-channel epineural interface will possibly be related to anatomy (e.g. surface area and curvature of the DRG) and stimulation safety (e.g. charge density limits) rather than electrode properties.

Finally, a fundamental assumption underlying these experiments is that selective recruitment of distal nerve branches corresponds to focal percepts localized to the limb. However, it is possible that the salience of an evoked percept is more relevant than focality for a somatosensory neural interface. Stimulation parameters that recruit a nerve may not necessarily evoke a percept and coactivation and non-selectivity may be permissible if evoked percepts are differentiable. However, addressing the subjective modality of percepts evoked via DRG stimulation may only be possible by replicating epineural DRG stimulation in humans. In summary, epineural electrodes represent a compromise between selectivity, safety, and stability and have been used in several successful neuromodulation devices. The selectivity of epineural stimulation at the DRG represents a viable path forward for clinical translation for a DRG-based somatosensory neuroprosthesis.

## Supplementary Material

Supplementary material

## Figures and Tables

**Table 1.** Epineural stimulation binary search parameters. ‘Total Electrodes’ represents the number of electrodes that were tested, ‘Active Electrodes’ represents the number of electrodes that were capable of recruiting any nerve at any charge. ‘Selective Electrodes’ represents the number of electrodes that recruited a single nerve at threshold. The binary search was conducted independently at 2 threshold resolutions for cat G at the L5 DRG.

Subject	Threshold resolution (nC)			Maximum charge (nC/phase)	Total electrodes	Active electrodes	Selective electrodes
	L5	L6	L7				
G	0.08, 0.82	—	0.21	16.38, 15.35	20	10	6
H	0.41	0.41	0.41	16.38	24	23	16
I	1.02	1.02	1.02	61.43	12	11	8
J	0.4	0.4	0.4	28.0	12	8	5
Total					68	52	35

**Table 2.** Penetrating stimulation binary search parameters. The binary search was conducted independently at 2 threshold resolutions for cat G at the L7 DRG.

Subject	Thieshold resolution (nC)			Maximum charge (nC/phase)	Total electrodes	Active electrodes	Selective electrodes
	L5	L6	L7				
E	—	0.20	0.20	3.07	192	61	49
F	—	0.20	0.20	8.19	256	38	28
G	0.08	0.12	0.12, 0.41	2.45	128	123	96
H	0.25	0.25	0.25	3.27	96	86	67
Total					672	308	240
