# Supplementary material for: Selectivity of afferent microstimulation at the DRG using epineural and penetrating electrode arrays
Source: J Neural Eng. Author manuscript; Available in PMC 2022 May 25. (PMC9131467; doi:10.1088/1741-2552/ab4a24)
Supplement: Supplementary material [file NIHMS1797751-supplement-Supplementary_material.pdf]

Supplementary material

Supplementary table 1: Post-implant nerve cuff motor thresholds. Nerve identities were determined using known anatomical landmarks and verified by finding coarse motor thresholds using a voltage-controlled stimulator.

	Nerve cuff	Motor Threshold (V)			
		G	H	I	J
**Femoral branch**	Femoral contact 2	0.24	0.36	0.32	0.25
	Femoral contact 4	0.24	0.36	0.48	0.38
	Saphenous	20	6	40	30
	Vastus Medialis	15	0.28	0.27	<0.1
	Vastus Lateralis	0.22	1	-	-
	Sartorius	24	50	0.23	0.4
**Sciatic Branch**	Sciatic contact 2	0.5	0.33	0.3	0.31
	Sciatic contact 4	0.65	0.28	0.36	0.26
	Tibial	0.29	12	0.1	0.18
	Medial Gastrocnemius	0.25	0.14	0.12	0.12
	Lateral Gastrocnemius	0.18	0.15	0.1	0.8
	Distal Tibial	0.18	0.17	0.16	0.28
	Common Peroneal	0.15	18	1	0.21
	Distal Common Peroneal	16	21	0.26	0.32
	Biceps Femoris	27	18	-	-
	Sural	no response	0.26	3.5	4.1

Supplementary table 2: Accuracy of the automated ENG detection algorithm per nerve cuff per epineural stimulation subject. Accuracy was calculated from true positive (TP), true negative (TN), false positive (FP), and false negative (FN) rates using the formula: (TP+TN)/(TP+TN+FP+FN) where an expert reviewer's manual annotations were used as ground truth for detection of ENG responses.

	G	H	I	J
**Femoral Nerve**	52.34	76.96	73.94	95.32
**Saphenous**	42.02	89.77	89.94	62.79
**Vastus Lateralis**	46.39	85.36	-	-
**Vastus Medialis**	45.60	92.34	86.23	77.47
**Sartorius**	37.71	82.50	90.17	93.79
**Sciatic Nerve**	94.27	96.99	100.00	99.19
**Tibial**	97.12	97.76	97.62	76.97
**Lateral Gastrocnemius**	93.67	96.79	100.00	97.49
**Medial Gastrocnemius**	96.30	95.80	99.40	93.82
**Distal Tibial**	96.40	98.23	100.00	92.44
**Common Peroneal**	93.33	97.17	91.43	54.84
**Distal Common Peroneal**	88.52	98.11	88.22	79.88
**Sural**	100.00	-	-	92.90
**Lateral Cutaneous**	-	94.50	99.42	74.86

**Medial Cutaneous**	-	94.56	99.43	-

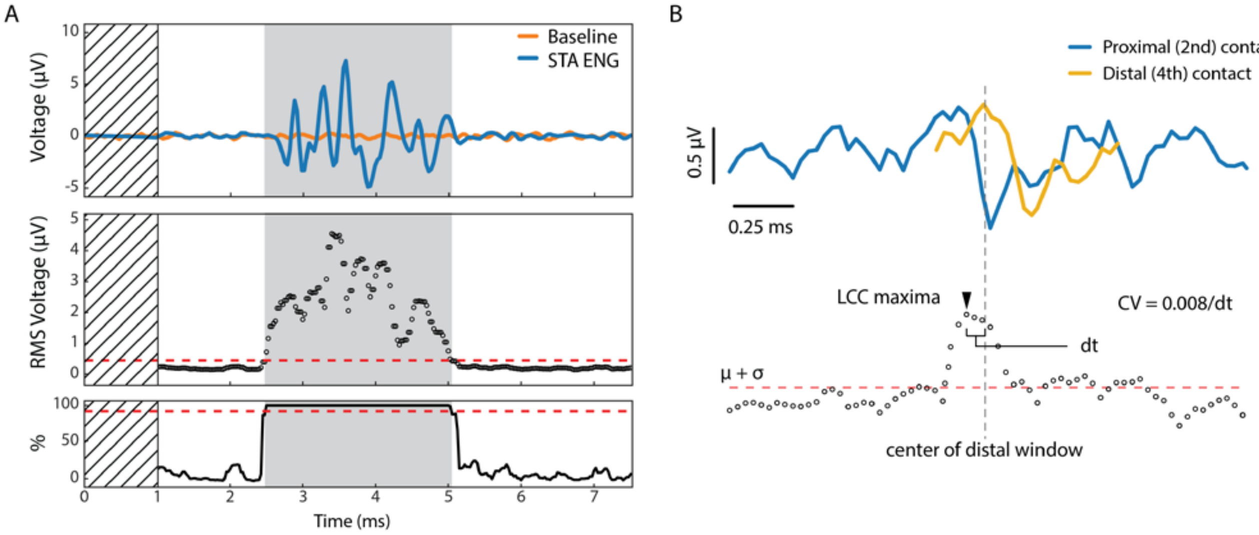

Supplementary figure 1: A) Example of automated ENG detection for a single trial. Top panel shows the stimulus triggered average ENG from the common peroneal nerve (blue) compared to the baseline ENG. Hatched section denotes 1 ms blanking period for stimulation artifact. Middle panel displays the average windowed RMS calculated using a 250 µs sliding window with 25 µs overlap between consecutive windows. Red dashed line indicates 99% confidence interval. Bottom panel shows the percentage of the subsampled averages that were supra-threshold (95%). B) Example of the local cross correlation calculated between ENG recorded on the 2nd (blue) and 4th (yellow) contact of the sciatic nerve cuff. Maximum of LCC is used to calculate the conduction velocity.

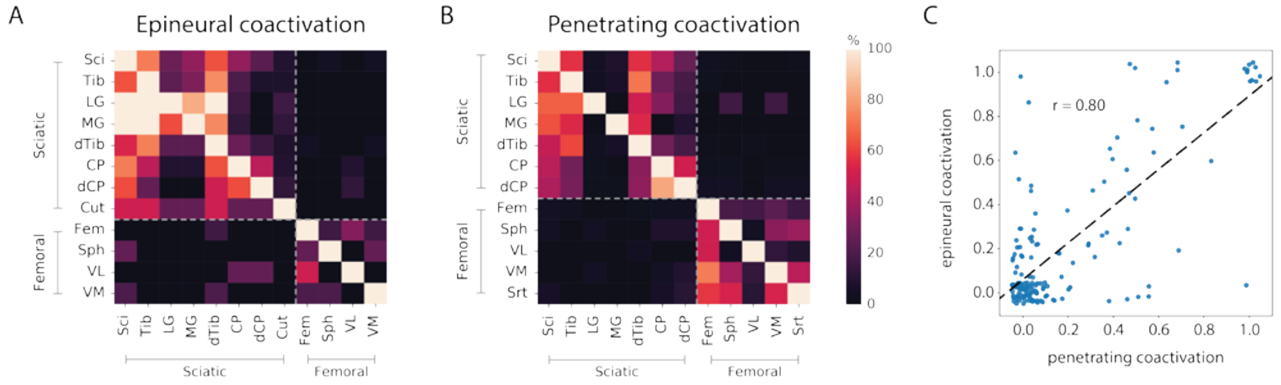

Supplementary figure 2: Normalized coactivation matrix for A) epineural and B) penetrating stimulation calculated by adding the coactivation matrix at each DRG and normalizing the counts in each row by dividing by the total number of times that a given nerve was recruited. C) Relationship between epineural and penetrating coactivation where each dot in the scatter plot represents one active-coactive nerve pair

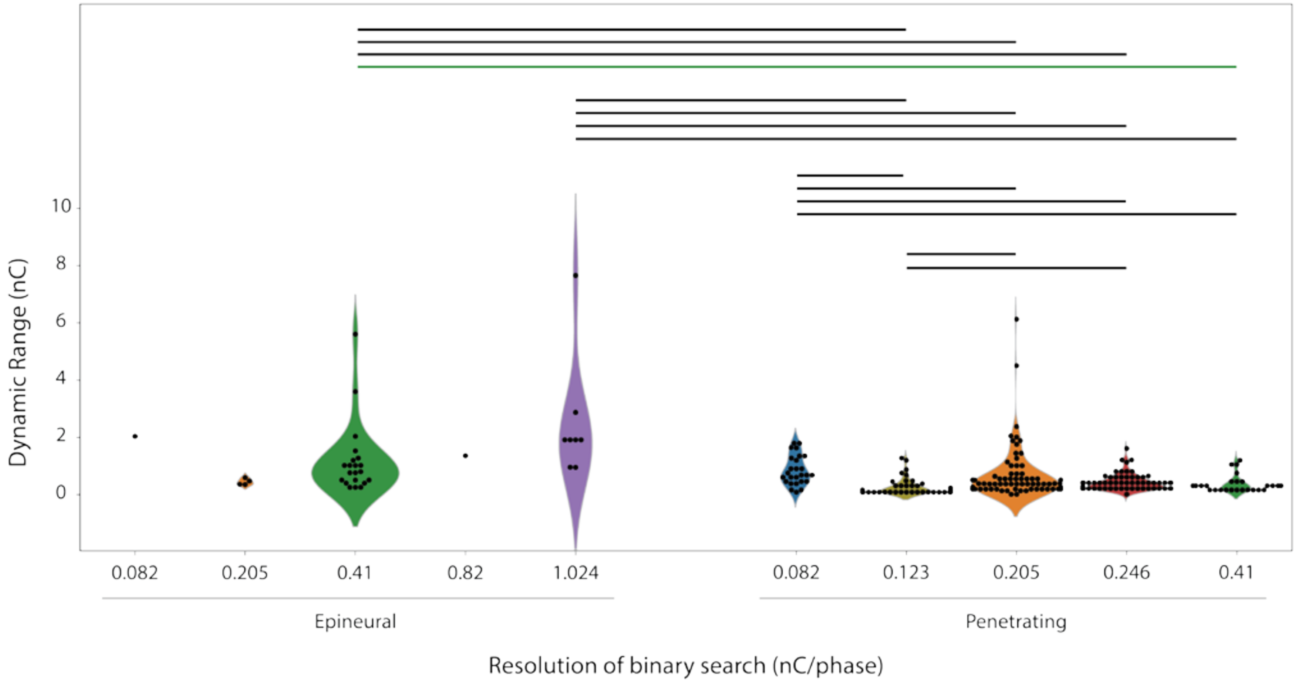

Supplementary figure 3: Distribution of dynamic ranges for each resolution of binary search and electrode type. Horizontal lines indicate distributions with significant differences using Dunn's nonparametric comparisons for post hoc Kruskal-Wallis testing
